# Supplementary material for: Using fractal model and factor analysis for FACA modeling and its application in deep mineral prediction
Source: PLoS One. 2026 May 8;21(5):e0348494. doi: 10.1371/journal.pone.0348494 (PMC13155623; doi:10.1371/journal.pone.0348494)
Supplement: Attachment — Submitted filename: Response to Reviewers.doc [file pone.0348494.s002.doc]

Dear Editor：

We would like to thank the reviewers and the editor for the positive and constructive comments and suggestions. We have revised our manuscript after reading the comments provided by the reviewers.

Answers to the Journal Requirements

1. Please ensure that your manuscript meets PLOS ONE's style requirements, including those for file naming.

**Answers:** We have revised the format of our manuscript as required in the revised version.

2. We note that the grant information you provided in the ‘Funding Information’ and ‘Financial Disclosure’ sections do not match. When you resubmit, please ensure that you provide the correct grant numbers for the awards you received for your study in the ‘Funding Information’ section.

**Answers:** We have confirmed the fund information in the cover letter.

3. Thank you for stating the following financial disclosure: “The author(s) received no specific funding for this work.” Please include your amended statements within your cover letter; we will change the online submission form on your behalf.

**Answers:** We have amended statements in the cover letter.

4. Thank you for stating the following in the Acknowledgments Section of your manuscript: “The authors are grateful to the Hubei Geological Bureau for providing the study data. This study was supported by the Opening Fund of Geomathematics Key Laboratory of Sichuan Province (Grant no. scsxdz2022-06), and the Program of Sichuan Mineral Resources Research Center (Grant no. SCKCZY2023-ZC006).” We note that you have provided funding information that is not currently declared in your Funding Statement. However, funding information should not appear in the Acknowledgments section or other areas of your manuscript. We will only publish funding information present in the Funding Statement section of the online submission form. Please remove any funding-related text from the manuscript and let us know how you would like to update your Funding Statement. Currently, your Funding Statement reads as follows: “The author(s) received no specific funding for this work.”

Please include your amended statements within your cover letter; we will change the online submission form on your behalf.

**Answers:** We have removed Funding from the revised manuscript and amended statements in our cover letter.

5. Thank you for stating the following in your Competing Interests section: “All of the authors have read the journal's policy and also had no conflict of interests.” Please complete your Competing Interests on the online submission form to state any Competing Interests. If you have no competing interests, please state "The authors have declared that no competing interests exist.", as detailed online in our guide for authors at http://journals.plos.org/plosone/s/submit-now

This information should be included in your cover letter; we will change the online submission form on your behalf.

**Answers:** We have done it in the cover letter.

6. Please note that your Data Availability Statement is currently missing the the DOI/accession number of each dataset or a direct link to access each database. If your manuscript is accepted for publication, you will be asked to provide these details on a very short timeline. We therefore suggest that you provide this information now, though we will not hold up the peer review process if you are unable.

**Answers:** We have done it the revised version.

7. When completing the data availability statement of the submission form, you indicated that you will make your data available on acceptance. We strongly recommend all authors decide on a data sharing plan before acceptance, as the process can be lengthy and hold up publication timelines. Please note that, though access restrictions are acceptable now, your entire data will need to be made freely accessible if your manuscript is accepted for publication. This policy applies to all data except where public deposition would breach compliance with the protocol approved by your research ethics board. If you are unable to adhere to our open data policy, please kindly revise your statement to explain your reasoning and we will seek the editor's input on an exemption. Please be assured that, once you have provided your new statement, the assessment of your exemption will not hold up the peer review process.

**Answers:** We have shared the study data at <https://github.com/qflong1/Studying-data.git>.

8. We notice that your supplementary figures and tables are included in the manuscript file. Please remove them and upload them with the file type 'Supporting Information'. Please ensure that each Supporting Information file has a legend listed in the manuscript after the references list.

**Answers:** We have removed figures and tables in the revised version, and upload them with the file type 'Supporting Information'.

9. If the reviewer comments include a recommendation to cite specific previously published works, please review and evaluate these publications to determine whether they are relevant and should be cited. There is no requirement to cite these works unless the editor has indicated otherwise.

**Answers:** There is no such thing.

10. Please review your reference list to ensure that it is complete and correct. If you have cited papers that have been retracted, please include the rationale for doing so in the manuscript text, or remove these references and replace them with relevant current references. Any changes to the reference list should be mentioned in the rebuttal letter that accompanies your revised manuscript. If you need to cite a retracted article, indicate the article’s retracted status in the References list and also include a citation and full reference for the retraction notice.

**Answers:** We have verified that there is no such thing.

Answers to comments

3. Have the authors made all data underlying the findings in their manuscript fully available?
The [PLOS Data policy](http://www.plosone.org/static/policies.action#sharing) requires authors to make all data underlying the findings described in their manuscript fully available without restriction, with rare exception (please refer to the Data Availability Statement in the manuscript PDF file). The data should be provided as part of the manuscript or its supporting information, or deposited to a public repository. For example, in addition to summary statistics, the data points behind means, medians and variance measures should be available. If there are restrictions on publicly sharing data—e.g. participant privacy or use of data from a third party—those must be specified.

**Answers:** We have shared the study data at <https://github.com/qflong1/Studying-data.git>. The data points behind means, medians and variance measures are showed in Table 2. We have uploaded them with the file type 'Supporting Information'.

5. Review Comments to the Author

Reviewer #1: The manuscript “Using fractal model and factor analysis for FACA modeling and its application in deep mineral prediction” presents a combined FACA approach for geochemical anomaly extraction and mineral prediction. The study is well-motivated, the methodology is clearly described, and the case study in the Jiguanzui mining area provides convincing evidence of the effectiveness of the proposed model. The integration of the concentration–area (C–A) model with factor analysis (FA) is appropriate and novel in application. The results are consistent with known ore bodies and provide plausible predictions of potential blind ore bodies. The conclusions are supported by the evidence, and the manuscript is overall well-structured and informative.
 One important issue concerns data availability. The current statement indicates that data may be obtained from the corresponding author upon request. This arrangement does not fully comply with the PLOS ONE Data Policy, which requires deposition in an open and unrestricted public repository. I encourage the authors to deposit the relevant datasets and analysis scripts in a recognized repository (e.g., Zenodo, OSF, Figshare) and provide persistent links in the revised manuscript to ensure compliance.
 Overall, the work is technically sound and suitable for publication, provided that the data availability issue is addressed. I recommend acceptance pending minor revision to ensure full compliance with data-sharing requirements.

**Answers:** We have shared the study data at <https://github.com/qflong1/Studying-data.git>.

Reviewer #2: The paper combines the C - A model and the FA method to design a new model FACA, which is used to determine the anomaly values of geochemical elements, thereby improving the accuracy of deep mineral prediction. The paper selects a representative specific study area to verify the application effect of the model. The processing of geochemical data in the study area is rigorous, and the conclusions are consistent with the actual situation of the study area, which can guide further mineral resource exploration in the study area. The paper has a clear logic, an innovative model, and supported conclusions. It is recommended to accept this paper. However, some language expressions in the paper need further correction. For example, in line 18, "model named the FACA for mineral prediction" is suggested to delete "the".

**Answers:** We have revised language expressions in the revised version.

Sincerely yours,

Feilong Qin, Hongjin Zhu, Yu Feng and ShiCheng Yu,

Thanks a lot.

Best regards
