# Supplementary material for: Using fractal model and factor analysis for FACA modeling and its application in deep mineral prediction
Source: PLoS One. 2026 May 8;21(5):e0348494. doi: 10.1371/journal.pone.0348494 (PMC13155623; doi:10.1371/journal.pone.0348494)
Supplement: Attachment — Submitted filename: Response to Reviewers.docx [file pone.0348494.s003.docx]

Dear Editor：

We would like to thank the reviewers and the editor for the positive and constructive comments and suggestions. We have revised our manuscript after reading the comments provided by the reviewers.

Answers to the Journal Requirements

1. I did not see the abstract of the paper, which is an indispensable component. The abstract should specifically highlight the fractal methodology used in this study and its application results.

**Answers:** This article has an abstract section, which is written in accordance with the format required by the journal.The abstract content is is as follows:

# Abstract

## Objective

This paper designs a FACA model for deep mineral prediction in actual mining areas.

## Method

The characteristics of geochemical anomalies that formed mineral resources and found the spatial distribution of geochemical anomalies was consistent with the concentration--area (C–A). Thus, this paper established a C–A model for geochemical anomaly extraction. Based on mineral resources formed by multiple element combinations, factor analysis (FA) was used to obtain the different combinations and comprehensive information of elements. On this basis, this paper designed a new model named FACA for mineral prediction using the FA and C–A.

## Results

The proposed FACA model was applied to mineral prediction in the Jiguanzui copper-gold mining area in China. The Ba, As, Ni, W, Au, Mo, Cu, and Ag elements in the study area were divided into four combinations: {W and Mo}, {Ba and Ni}, {As}, and {Au, Cu, and Ag} by using FACA model. The thresholds of Ba, As, Ni, W, Au, Mo, Cu, and Ag were 398.3384, 25.3168, 19.1416, 16.7271, 79.7408, 28.7244, 896.1419, and 0.6393, respectively, the thresholds of factor scores for {W and Mo}, {Ba and Ni}, {As}, and {Au, Cu, and Ag} were 0.4321, 0.6363, 0.3943, and 0.2958, respectively. The threshold of the comprehensive information of the eight elements was 0.2958. Through diagnostic testing, the abnormal distributions of geochemical elements were consistent with their theoretical distributions, and the comprehensive abnormal distribution area of the eight elements was consistent with the distribution of the actual ore bodies, demonstrating that the designed FACA algorithm of this paper was reasonable.

## Conclusions

A new blind ore body in the study area is predicted by using FACA model, positioned at a depth ranging from approximately 1120m to 1150m below ground, between drill holes ZK02618 and KZK23. These findings hold significant implications for mineral exploration efforts.

2. Significant research has been conducted on fractal theory in deep mineral exploration and development, which is not reflected in the Introduction. It is essential to include the latest advancements related to fractal theory and mineral exploration, with reference to works such as: https://doi.org/10.1021/acs.energyfuels.4c03095 and https://doi.org/10.62762/JGEE.2025.365363. The literature review should not be limited solely to these references.

**Answers:** We have added relevant fractal theory literature in the revised version.

3. The quality of the figures is relatively poor, with some data points being unclear. Please standardize and adjust the figures accordingly.

**Answers:** We have done it in the revised version.

4. The figures should be embedded within the main text of the manuscript. Otherwise, it is challenging for reviewers to cross-reference them during evaluation.

**Answers:** After adding figures to the manuscript and resubmitting them, the journal asked us to remove them from the manuscript.

The requirements for the journal are as follows

“PONE-D-25-39619R2

Using fractal model and factor analysis for FACA modeling and its application in deep mineral prediction

Professor Feilong Qin

Dear Dr. Qin,

We've checked your submission and before we can proceed, we need you to address the following issues:

1. We note that there were multiple versions of Manuscript in your submission's file inventory. We have removed the older file(s), retaining the most recent version(s) for editorial review. Please double check your submission file inventory and let us know if any files appear to be outdated or absent.

2. Please remove your figures/ from within your manuscript file, leaving only the individual TIFF/EPS image files. These will be automatically included in the reviewer’s PDF

3. Please include your tables as part of your main manuscript and remove the individual files. Please note that supplementary tables (should remain/ be uploaded) as separate "Supporting Information" files

4. We notice that your Figures are uploaded with the file type 'Supporting Information'. Please amend the file type to 'Figure'.

Thank you for submitting your work to PLOS ONE and supporting our mission of Open Science.

Kind regards,

Trisha Mae Tañedo Perez

PLOS One”

5. Please do not use comment/annotation mode. Instead, incorporate the revisions directly into the text, highlight the changes in red, and upload this version solely as a Supplementary Material file.

**Answers:** We have done it.

6. Finally, please standardize the formatting of the manuscript, particularly the references. The current format does not conform to the journal's publication requirements.

**Answers:** We have done it in the revised version.

Sincerely yours,

Feilong Qin, Hongjin Zhu, Yu Feng and ShiCheng Yu,

Thanks a lot.

Best regards
