# Supplementary material for: Using fractal model and factor analysis for FACA modeling and its application in deep mineral prediction
Source: PLoS One. 2026 May 8;21(5):e0348494. doi: 10.1371/journal.pone.0348494 (PMC13155623; doi:10.1371/journal.pone.0348494)
Supplement: Attachment — Submitted filename: Response_to_Reviewers_auresp_3.docx [file pone.0348494.s004.docx]

Dear Editor：

We would like to thank the reviewers and the editor for the positive and constructive comments and suggestions. We have revised our manuscript after reading the comments provided by the reviewers.

**Answers to the Journal Requirements**

1. If the reviewer comments include a recommendation to cite specific previously published works, please review and evaluate these publications to determine whether they are relevant and should be cited. There is no requirement to cite these works unless the editor has indicated otherwise.

**Answers:** We have checked it.

2. Please review your reference list to ensure that it is complete and correct. If you have cited papers that have been retracted, please include the rationale for doing so in the manuscript text, or remove these references and replace them with relevant current references. Any changes to the reference list should be mentioned in the rebuttal letter that accompanies your revised manuscript. If you need to cite a retracted article, indicate the article’s retracted status in the References list and also include a citation and full reference for the retraction notice.

**Answers:** We have checked the references and added 2 references in the revised manuscript.

**Answers to Additional Editor Comments**

1. The quality of the figures is too poor, and their formatting is not standardized. I recommend revising them one by one.

**Answers:** We have revised figures.

2. Please insert the figures and tables into the main text and ensure that each corresponds to the content accordingly.

**Answers:** The journal requires the removal of figures from the manuscript. “Leaving only the individual TIFF/EPS image files. These will be automatically included in the reviewer's PDF.”

3. The abstract is too lengthy, particularly the methods and results sections, which can be condensed.

**Answers:** We have simplified the text of the abstract.

4. The introduction does not sufficiently reflect the current state of fractal model and factor analysis for FACA modeling. In particular, the authors should emphasize how this study differs from previous research, thereby further highlighting the novelty of the paper.

**Answers:** We have added relevant content in the introduction section, as shown in the revised draft.

5. The ideal manuscript structure is: 1. Introduction, 2. Materials and Methods, 3. Results, 4. Discussion, followed by Conclusions. All sections and subsections must be numbered properly, e.g., 1. Introduction, 2. ...

**Answers:** We wrote the manuscript in the format required by the PLOS ONE journal.

6. Please pay appropriate attention to some of the papers already published in the journal and further increase the number of references, such as papers on fractal prediction methods (https://doi.org/10.1007/s40948-026-01117-7).

**Answers:** We have added relevant fractal-related literature, as shown in the revised manuscript.

7. Finally, please strictly follow the journal's formatting guidelines when preparing the manuscript.

**Answers:** Our revised manuscript has been formatted according to the PLOS ONE journal 's requirements.

Sincerely yours,

Feilong Qin, Hongjin Zhu, Yu Feng and ShiCheng Yu,

Thanks a lot.

Best regards
